# Seismic Performance Evaluation of RC Columns Retrofitted by 3D Textile Reinforced Mortars

**DOI:** 10.3390/ma15020592

**Published:** 2022-01-13

**Authors:** Siyun Kim, Sung Jig Kim, Chunho Chang

**Affiliations:** 1 Department of Architectural Engineering, Keimyung University, Daegu 42601, Korea; kimsiyun1989@gmail.com; 2 Department of Civil Engineering, Keimyung University, Daegu 42601, Korea; chunho@kmu.ac.kr

**Keywords:** textile reinforced mortar, 3D textile, rectangular RC column, retrofitting method, seismic performance

## Abstract

The paper investigates the seismic performance of rectangular RC columns retrofitted by a newly developed 3D Textile Reinforced Mortar (TRM) panel. The 3D-TRM used in this study consists of two components: self-leveling mortar and 3D textiles. Firstly, the flexural capacity of the 3D-TRM panel was investigated through the four-point flexural test. Secondly, a total of five specimens were constructed and experimentally investigated through static cyclic loading tests with constant axial load. One specimen was a non-seismically designed column without any retrofit, while the others were strengthened with either the 3D-TRM panel or conventional Fiber Reinforced Polymer (FRP) sheets. Experimental results in terms of hysteretic behavior, ductility ratio, and energy dissipation are investigated and compared with the cases of specimens with conventional retrofitting methods and without any retrofit. The maximum lateral force, ductility, stiffness degradation, and energy dissipation of RC columns with 3D-TRM panels were significantly improved compared with the conventional RC column. Therefore, it is concluded that the proposed retrofitting method can improve the seismic performance of non-conforming RC columns.

## 1. Introduction

The seismic vulnerability of non-conforming RC buildings, coupled with their aging and deterioration, is apparent from recent experiences with large earthquakes such as the earthquake in Haiti (Mw 7.0, 2010), the Maule earthquake in Chile (Mw 8.8, 2010), and the Sichuan earthquake in China (Mw 7.9, 2008) [1,2,3]. These buildings are designed either without any seismic details or with inadequate details of the past seismic design codes. Most of them are aged and thus suffer from the extensive deterioration that affects their durability. Karapetrou et al. (2017) [4] evaluated the seismic vulnerability of RC frames by considering the aging effect. This study confirms that the seismic vulnerability over time increases due to the corrosion related to aging effects on structural behavior. The reconnaissance reports [5] on the Pohang earthquake in South Korea (Mw 5.4, 2017) confirm that construction quality defects could cause significant damage to modern RC buildings. In particular, columns of many piloti-type low-rise RC buildings were severely damaged due to poor construction practices. The significant damage of many RC columns, which were thought to be seismically designed, was observed due to excessive concrete cover, the inclusion of drainage pipes in the column members, the inadequate spacing of the shear reinforcement, or the poor anchorage of ties. Kuang et al. (2005) [6] experimentally investigated the behavior of non-seismically designed RC columns with various configurations of shear reinforcement. Dang et al. (2017) [7] also experimentally evaluated the effects of biaxial bending moment and axial force on RC columns with various stirrup spacings. In particular, the specimens were similar the corner columns of piloti-type RC buildings that suffered significant damage from the Pohang earthquake. The experimental results showed that the failure mode of an RC column could be altered due to stirrup spacing. Kim et al. (2021) [8] evaluated the seismic vulnerability of RC frames by considering the uncertainties of material strengths and section properties due to the possible construction quality defects and aging. It was concluded that variability in concrete strength and the volumetric ratio of the transverse reinforcement mainly affected the seismic vulnerability of RC frames.

Many recent researches on retrofitting methods to improve the seismic performance of such structures described above have focused on the application of Fiber Reinforced Polymers (FRP) [9,10,11,12]. Katsumata et al. (1988) [9] experimentally evaluated the seismic performance of RC columns strengthened by carbon fiber sheets. Ozcan et al. (2008) [10] conducted static cyclic loading tests for RC columns by considering the number of layers of carbon FRP and the presence and absence of the axial load on the column. The test results were compared with the analytical results with a simple plastic hinge model considering the effects of FRP confinement and slip of plain bars. Lee et al. (2012) [11] evaluated the experimental performance of RC columns with aramid FRP sheets and strips. As a result, the strength and energy dissipation of RC columns retrofitted by aramid FRP sheets were superior to those of the specimens retrofitted by aramid FRP strips. Kim et al. (2014) [12] evaluated the seismic performance of RC columns using the developed FRP composite for rapid retrofitting. The developed FRP composites were manufactured of laminating only glass fiber or a combination of aluminum plates with holes and glass fiber. The results indicate that shear demand and cumulative energy dissipation of the specimen with the combination of aluminum plate and glass fiber increased compared to specimen with laminating only glass fiber, while the ductility of them was reversed.

Despite many advantages, FRP has the disadvantage of high cost and vulnerability at high temperatures. Recently, Textile Reinforced Mortar (TRM) has been studied as a material of FRP alternative. The TRM is the composite material comprised of a cement-based matrix and continuous multifilament yarns, and thus has high performance including durability, lightweight, and high strength [13,14,15]. Many studies indicate that the structural performance of RC members strengthened by TRM has been improved [16,17,18,19,20,21,22]. Triantafillou et al. (2006) [17] conducted tests with the cylinders and short rectangular columns strengthened by TRM to investigate the effect of the retrofitting method on the axial force capacity. This experimental result showed that the axial force of the specimen retrofitted by TRM increased significantly. The axial force also increased as the number of layers of the textile increased. Brückner et al. (2006) [18] experimentally evaluated the bending and shear capacities of RC slabs and beams strengthened by Textile Reinforced Concrete (TRC) with fine-grained concrete. It was concluded that both the load-carrying and shear capacities increased compared with the reference specimen. Moreover, the ultimate load and serviceability of the RC member were improved.

In this study, a 3D-TRM panel is developed by combining 3D textiles with high tensile strength and mortars with excellent fluidity. The retrofitting method using 3D-TRM panels is proposed to improve the seismic performance of non-conforming RC columns. In addition, it is fabricated as a precast product to ensure uniform quality and shorten the construction period on site. The paper presents the details of the newly developed 3D-TRM panel and its retrofitting method. The experimental program is also described to evaluate the seismic performance of RC columns retrofitted by the 3D-TRM panel through cyclic loading tests. The observed lateral force, ductility, stiffness degradation, dissipated energy, and strain distribution of the retrofitted specimens are evaluated and compared with those of the non-retrofitted specimen.

## 2. 3D-TRM Panel

### 2.1. Proposed Method Using 3D-TRM Panel

**Figure 1:**
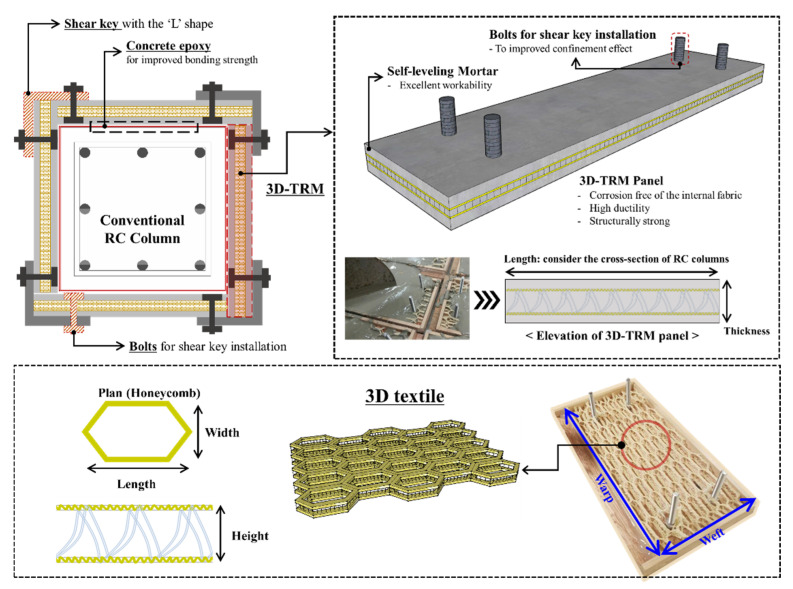
3D-TRM panel and retrofitting method.

In order to improve the seismic performance of RC columns, a 3D-TRM panel is developed as the composite material comprised of a cement-based matrix with fine aggregate and three-dimensional textiles with high performance. The configuration and details of the 3D-TRM retrofitting method are shown in Figure 1. The developed seismic reinforcements are precast products and can be manufactured with uniform quality. In addition, the retrofitting method using the proposed 3D-TRM panel can improve the workability and shorten the construction period as the prefabricated method and thus rapid retrofit of damaged structures right after an earthquake could be possible. The 3D-TRM panels are attached to the surface of conventional RC columns by using L-shaped shear keys and concrete epoxy as shown in Figure 1. To improve the confinement effect of 3D-TRM on the RC member, the shear key is fastened at the edge of the rectangular RC column.

### 2.2. Properties of 3D-TRM Panel

#### 2.2.1. Mortar and Textile

The mortar used for the 3D-TRM panel must be able to pass through the complex textile mesh without interference. However, 3D textiles are composed of two-layers grid textiles as shown in Figure 1. It is difficult for normal mortars to pass through the 3D textiles which have a low height between each layer and small grid size. Thus, the self-leveling mortar is selected for this study and has the characteristics of high fluidity and compressive strength similar to or higher than the conventional mortar. The 28-day compressive strength of the selected self-leveling mortar is 43.1 MPa.

Table 1 shows the mechanical properties of 3D textiles and conventional fabrics used for retrofitting RC columns in this study. As described previously, the developed 3D-TRM panels consists of the 3D textiles with two-layer mesh fabric and self-leveling mortar. The effects of the grid size and height between layers of the 3D textiles on the seismic performance of retrofitted members need to be evaluated. Thus, two 3D textiles are selected for this study, which are woven with the grid sizes and layer heights of 18 × 20 × 4 mm and 25 × 12 × 6 mm (length × width × height), respectively. In addition, the RC columns strengthened by conventional carbon FRP and aramid FRP sheets are also included in the experimental program. The tensile strengths and elongations of 3D textiles and conventional fabrics were investigated by carrying out the tensile testing based on KS K 0521 [23]. As detailed in Table 1, the tensile strengths of 3D textiles are significantly lower than those of conventional fabrics due to the low amount of yarn per unit area.

#### 2.2.2. Flexural Behavior of 3D-TRM Panel

The effects of layer height and grid size of 3D textiles on the flexural behavior of the developed 3D-TRM panel were investigated. As described in Table 2, three cases with and without 3D textiles were considered and three specimens for each case were constructed. The longitudinal direction of the specimen was parallel to the warp direction of textiles having the higher tensile strength as shown in Table 1. Thus, a total of nine specimens were constructed and tested through a four-point bending test by utilizing 5 MN compression tester (CCH-5000kN, Shimadzu in Japan) in Intelligent Construction System Core-Support Center at Keimyung University. The length, width, and height of the specimens for flexural test are 400 mm, 10 mm, and 30 mm, respectively.

**Figure 2:**
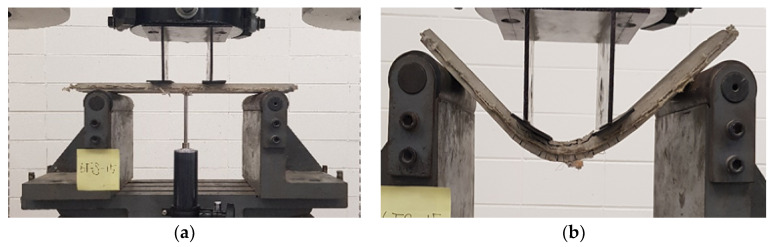
Flexural test with 3D-TRM panel: (**a**) Test setup; (**b**) End of test.

**Figure 3:**
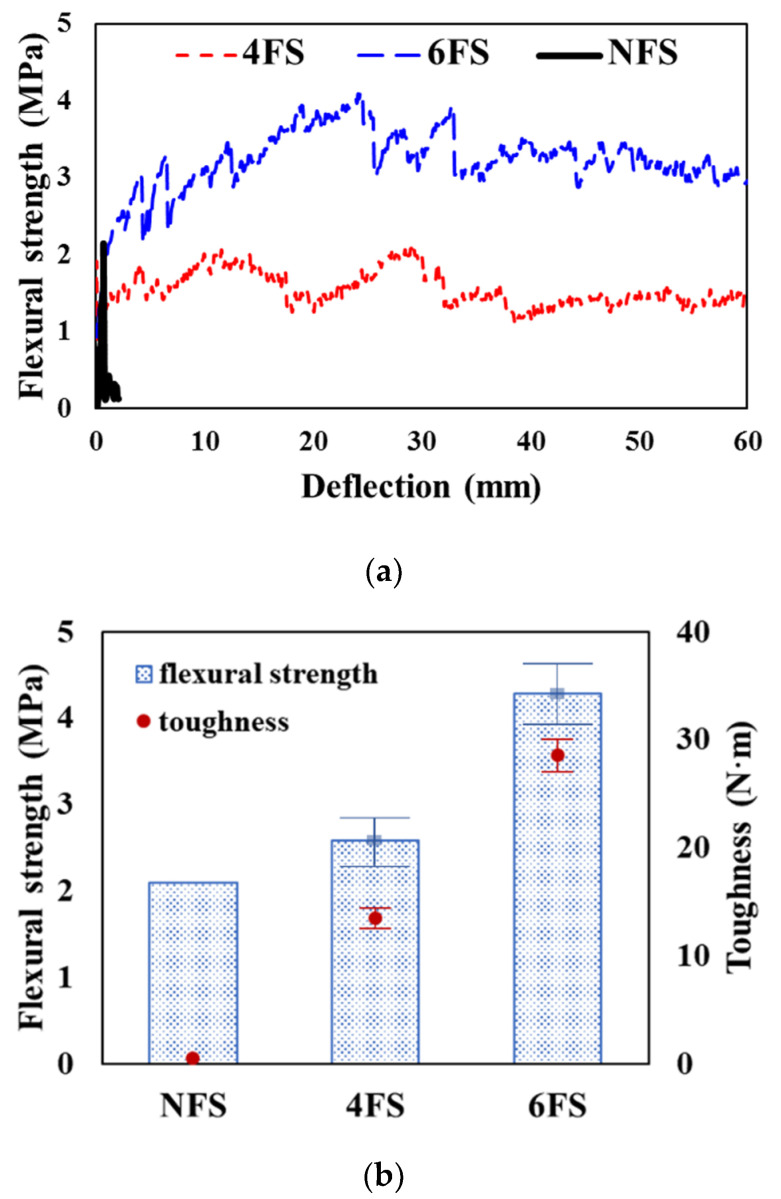
Flexural test results: (**a**) Flexural strength and deflection; (**b**) Flexural strength and toughness.

Figure 2 shows the test setup and substantial ductile behavior of the 3D-TRM panel. The specimen NFS showed the brittle failure with a small deflection, while the 3D-TRM panel did not suffer complete failure despite a significant deflection of more than 60 mm due to the effect of embedded 3D textiles. The experimental results for the flexural performance of specimens with or without 3D textiles are shown in Figure 3. The toughness shown in Figure 3 is calculated by taking the area enclosed by the corresponding relationship curve between the flexural strength and deflection. The flexural strengths of specimens with 3D textiles were linearly increased with the increase of the deflection until the first crack occurred. The first crack occurred in the pure bending stress zone between the upper two loading points. Once the first crack occurred, the specimens without 3D textiles failed immediately, while the flexural strengths of specimens with 3D textiles were fluctuated but remained until a significant amount of deflection occurred. The maximum flexural strength of specimens 4FS and 6FS in increased up to 19.1% and 104.8%, respectively, compared to specimen NFS. In particular, the flexural strength of specimen 6FS significantly increased after the first crack due to the effect of 3D textiles. The toughness of all specimens with 3D textiles significantly increased compared to non-retrofitted specimen. The toughness of specimen 6FS increased up to 92.1% compared with that of specimen 4FS. This is because, compared with specimen 4FS, the specimen 6FS has 3D textiles with larger spacing and thus, its grid fabric layer is closer to the tensile zone.

## 3. Experimental Program

### 3.1. Design and Details of Specimens

The experimental program aimed to investigate the effect of the proposed retrofitting method using the 3D-TRM panel on the seismic performance of non-conforming RC columns. Thus, a total of five specimens with a scale factor of 1/3 were designed with non-seismic details and constructed. The first specimen was non-retrofitted, while the rest of the specimens were strengthened with either the developed 3D-TRM panels or conventional FRP sheets. The conventional wrapping method for FRP sheets is used and its retrofitting procedure can be found elsewhere [24,25,26], while the retrofitting method using 3D-TRM panels is described in Section 3.2. The selected retrofitting types and properties of concrete and reinforcements for test specimens are detailed in Table 3.

**Figure 4:**
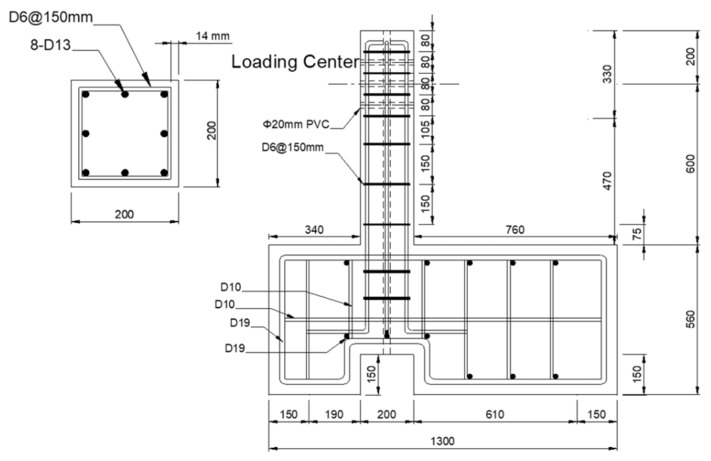
Section and elevation of test specimens (unit: mm).

As shown in Figure 4, the cross-section of test specimens is 200 × 200 mm with 8-D13 longitudinal rebars and D6 stirrups. The spacing of the transverse rebars is 150 mm. Thus, as detailed in Table 3, the longitudinal rebar ratio is 2.53% and the volumetric ratio of the transverse rebar is 0.51%, which indicates the low confinement. The desired concrete strength was 27 MPa, while the obtained compressive strength from cylinder tests was 24.7 MPa. The effective height of the column specimens is 600 mm, resulting in an aspect ratio of 3.0. Thus, these sparse transverse rebars and relatively low aspect ratio are expected to contribute to the shear failure of column specimens. The stiff end cap beam at the bottom of the column has length, width, and height of 1300 mm, 800 mm, and 560 mm, respectively, and is designed conservatively to avoid significant deformation and development of cracks during the tests.

### 3.2. Retrofitting Procedure Using 3D-TRM Panel

The development of a precast 3D-TRM panel is intended to ensure improved the seismic performance as well as the uniform quality control of retrofitted RC members. In addition, the prefabricated construction method using the precast products with uniform quality can shorten the construction period and thus, may rapidly strengthen inadequate RC members and improve their seismic performance after an earthquake. This retrofitting method consists of four steps. The first step is to remove the irregularities on the surface of the selected member. Secondly, 3D-TRM panels coated with epoxy are attached to the member. Thirdly, L-shaped shear keys are installed and fastened in order to strengthen the sectional edges of the rectangular member. The last step is to cure the concrete epoxy.

**Figure 5:**
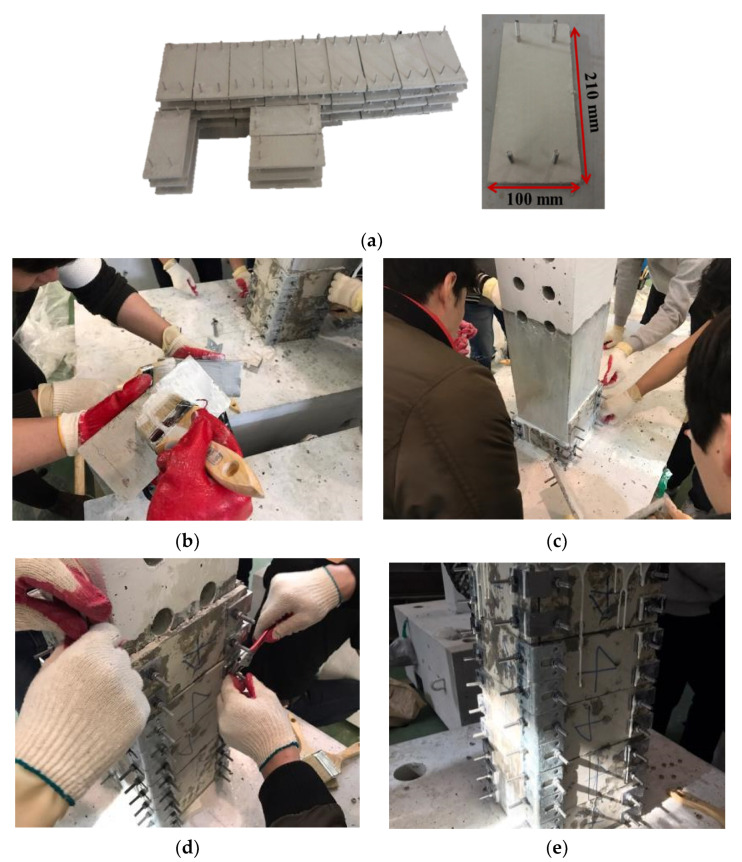
Retrofitting process of the specimen with 3D-TRM panel: (**a**) 3D-TRM panels; (**b**) Epoxy coating on the surface of 3D-TRM panel and RC column; (**c**) Attachment of 3D-TRM panel; (**d**) Installation of shear keys; (**e**) Curing epoxy.

The 3D-TRM panel manufactured in this study has length, width, and thickness of 210 mm, 100 mm, and 10 mm, respectively. Note that the longitudinal direction of the 3D-TRM panel is parallel to the warp direction of the textiles. Furthermore, textiles detailed in Table 1 are used to manufacture the 3D-TRM panel in this study. The thickness of the 3D-TRM panel is determined by considering the maximum gap between layers of the selected 3D textiles. Figure 5 illustrates the 3D-TRM panel used in this study and its retrofitting process.

### 3.3. Loading and Instrumentation Plan

**Figure 6:**
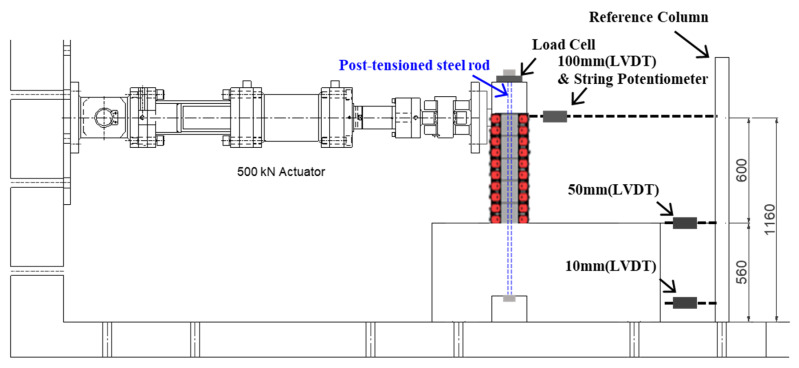
Test setup (unit: mm).

Figure 6 illustrates the schematic view of the test setup including a test specimen with 3D-TRM panels. The horizontal actuator was used to apply lateral forces and the vertically post-tensioned steel rod was responsible for the compression axial load.

Strain gauges, Linear Variable Displacement Transducers (LVDT), and a string potentiometer were utilized to measure the strains of longitudinal and transverse rebars and lateral displacements of the specimen during the experiment. As shown in Figure 6, the LVDT and string potentiometer were used to measure the displacement at the loading point. Two LVDTs were used to monitor the slippage and rotation of the cap beam at the bottom of the test specimen.

**Figure 7:**
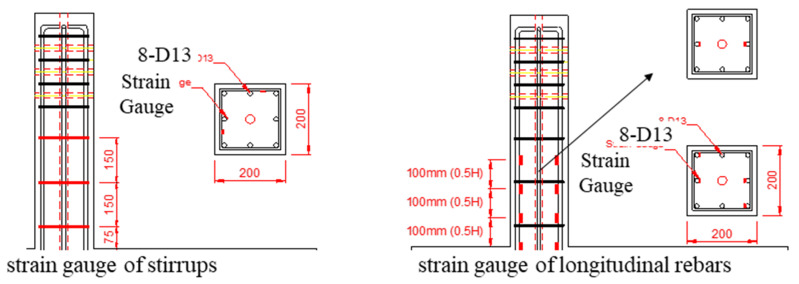
Strain gauges.

Figure 7 indicates the location of strain gauges for longitudinal and transverse rebars. Four layers of strain gauges on longitudinal bars were placed by considering the possible plastic hinge area. Four longitudinal bars per layer had a gauge except for the top layer. Each transverse bar had two gauges attached on its perimeter. Thus, a total of 20 strain gauges were attached to the longitudinal rebars and stirrups.

**Figure 8:**
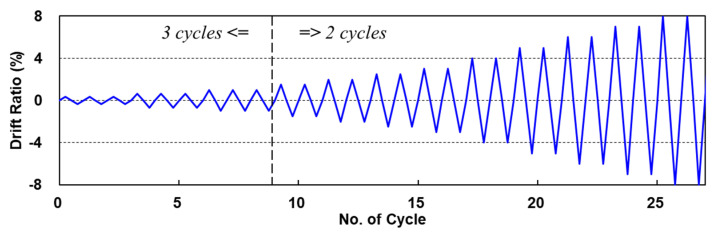
Loading history.

Figure 8 shows the applied lateral displacement history for the static cyclic tests with a constant axial load. The applied initial compressive axial force is 108 kN which is 10% of the column capacity ($0.1f_{c}^{\prime }A_{g}$), where $f_{c}^{\prime }\;$ is the compressive strength of the concrete, and $A_{g}$ is the sectional area of the RC column.

The imposed lateral displacement history includes three cycles at each displacement level up to a drift ratio of 1%, and two cycles at each displacement level after 1%. Multiple cycles at each displacement level are to reflect the effect of strength degradation characteristics.

## 4. Experimental Results

### 4.1. General Observations

**Figure 9:**
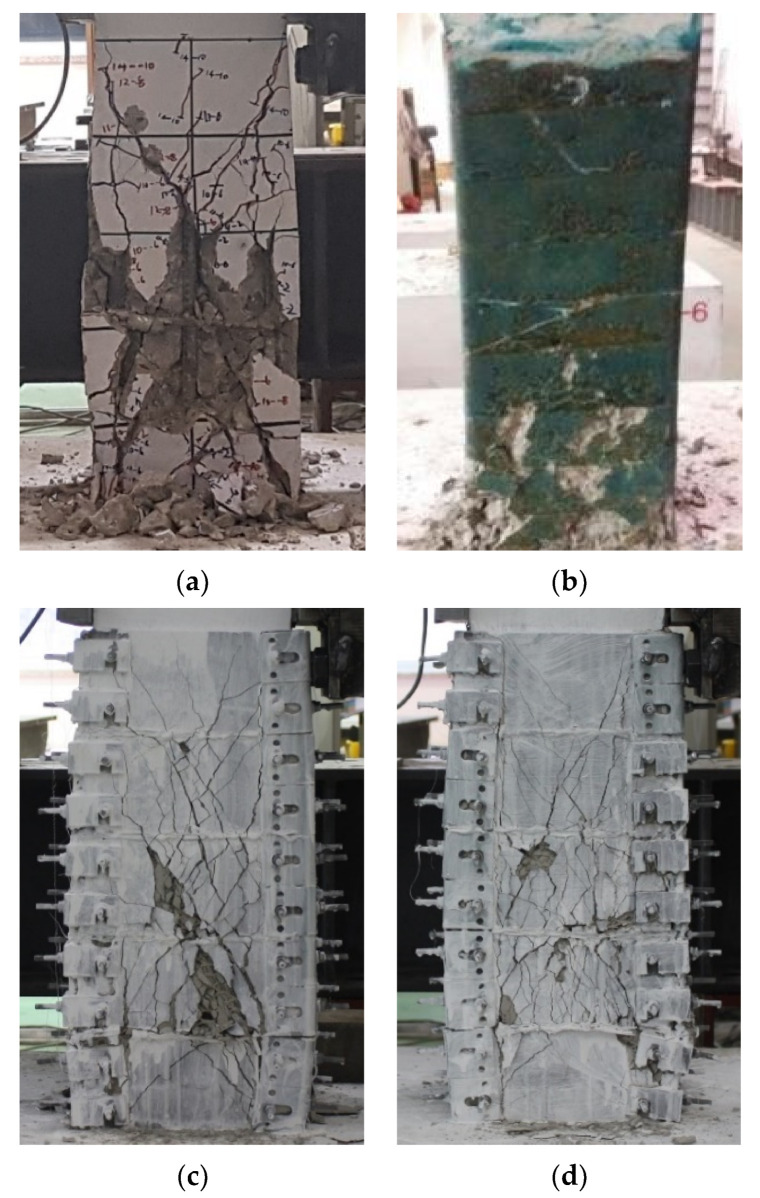
Crack patterns: (**a**) NRF; (**b**) ARF; (**c**) 3DRF4; (**d**) 3DRF6.

Figure 9 illustrates the cracking patterns of all specimens at the end of the tests. The crack patterns of specimens retrofitted by conventional FRP sheets were investigated after the experiments were finished and the FRP sheets were unwrapped. On the other hands, the cracks on the non-retrofitted specimen (NRF) and specimens retrofitted by the 3D-TRM panel were observed during the tests. The experiment was terminated at the drift ratio of either 6% or 7% due to the limitations of the actuator and measurements.

The initial cracks of the specimen NRF occurred at the drift ratio of 0.67%, and then X-shaped shear cracks were developed as the width of the crack increased. Furthermore, shear failure along with spalling damage at the plastic hinge zone and severe damage of core concrete were observed at a drift ratio of 2.56%. The specimens 3DRF4 and 3DRF6, retrofitted by the 3D-TRM panels, showed initially flexural behavior and inclined hair cracks were developed as shown in Figure 9. The strength of the specimen 3DRF6 was slightly dropped after the drift ratio of 3.68% with inclined cracks. In particular, the specimen 3DRF4 exhibited wider and severe diagonal cracks compared with those of the specimen 3DRF6. The specimens CRF and ARF, retrofitted with conventional FRP sheets, showed the typical flexural failure and had fewer cracks compared to those of the specimen NRF.

### 4.2. Hysteretic Behavior

**Figure 10:**
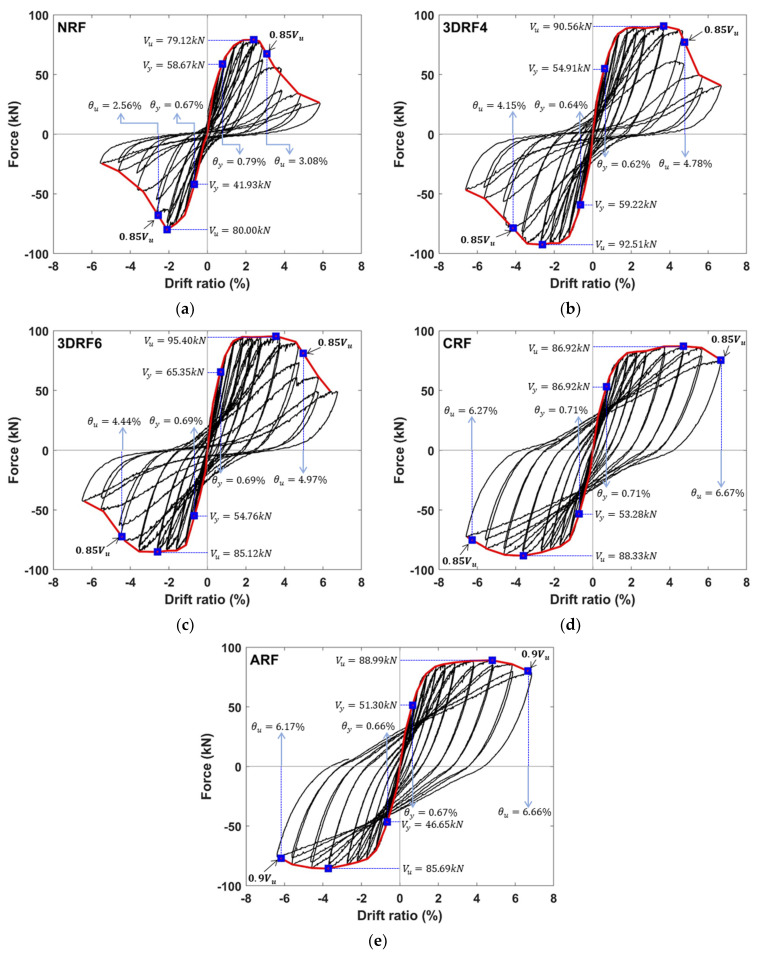
Lateral force and drift ratio: (**a**) NRF; (**b**) 3DRF4; (**c**) 3DRF6; (**d**) CRF; (**e**) ARF.

The relationship between the lateral force and drift ratio for all specimens is shown in Figure 10 and measured forces are detailed in Table 4. Each hysteretic curve in Figure 10 is marked with three fundamental forces on each branch: yield force ($V_{y}$), ultimate force ($V_{u}$), and force ($0.85V_{u}$) corresponding to the ultimate displacement. The yield force is assumed to be the force corresponding to the first yield point. The ultimate displacement is determined at the point of 15% drop in strength from the peak of the maximum applied load. Note that the force corresponding to the ultimate displacement of the specimen ARF is approximately 90% of the maximum force, when the experiment was terminated due to the limitation of testing facilities. The significant impact of the seismic retrofit on the seismic performance of RC columns can be clearly observed in Figure 10. The hysteretic curve of the specimen NRF shows a typical shear-critical behavior and relatively rapid strength degradation after reaching the drift ratio of 2.40%. The specimens 3DRF4 and 3DRF6 using the proposed retrofitting method show the flexural behavior around the drift ratio of up to 3.6% and afterward, notable strength degradation as shown in Figure 10b,c. In contrast, stable flexural hysteretic behavior is observed in the specimens CRF and ARF retrofitted with the conventional FRP sheets as shown in Figure 10d,e.

As shown in Table 4, the average of the measured maximum lateral forces in both directions is calculated and compared with that of the non-retrofitted specimen. Compared with the specimen NRF, lateral forces of specimens CRF and ARF increased up to 10.14% and 9.78%, respectively, while the lateral forces of specimens 3DRF4 and 3DRF6 increased up to 15.05% and 13.45%, respectively. Thus, the effect of the proposed retrofitting method using 3D-TRM panels on the lateral force of the test specimen is higher than that of the conventional FRP retrofitting method. It could be inferred that the sectional size of the RC column with 3D-TRM panels slightly increases due to the panel thickness and the higher confinement capacity of the RC member is ensured due to the applied 3D-TRM panels and shear keys. In addition, the lateral force of the specimen 3DRF4 increased up to 1.42% compared with that of the specimen 3DRF6 because of the slightly larger amount of fiber per unit area.

**Figure 11:**
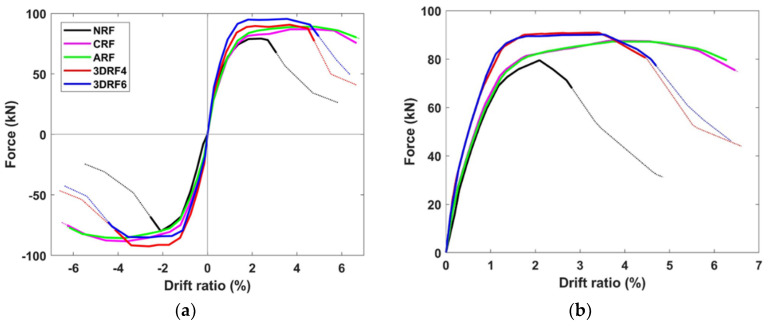
Envelope curve: (**a**) Both directions; (**b**) Average curve.

The envelope curves in both directions and their average envelope curve are shown in Figure 11. Compared with NRF, Figure 11b indicates clearly the notable improvement of stiffness, strength, and ultimate displacement of the specimens 3DRF4 and 3DRF6. Particularly, retrofitting effect of 3D-TRM panels on the stiffness and strength of the RC column is superior to that of FRP sheets. In addition, ultimate drift ratios of the specimens 3DRF4 and 3DRF6 are 4.46% and 4.70%, respectively, which is much larger than 2.56% of the specimen NRF. However, those are considerably less than ultimate drift ratios of specimens CRF and ARF. Dymiotis et al. (1999) [27] has derived a statistical distribution for the critical drift ratio using previous experimental results. The mean drift ratios obtained from the distribution are 4.0 and 6.6 for near failure and failure, respectively. Lu et al. (2005) [28] investigated probabilistic drift limits of RC columns and showed that the mean limits at the ultimate level for RC columns vary in 3.0–7.8%. Several values for the drift ratio for collapse limit have been suggested by seismic codes and guidelines. Emergency Management Agency (FEMA) limits the allowable drift ratio of 4% to collapse prevention [29], and the design codes limit the allowable drift ratio to 2% [30,31]. Thus, the ultimate drift ratio of the specimen with the proposed retrofitting method could be acceptable because it is much larger than allowable drift limit in seismic codes and within the range of drift ratios from previous studies. Therefore, the proposed retrofitting method can improve the strength as well as deformation capacity of seismically vulnerable RC columns.

### 4.3. Effect on Ductility

The ductility ratio of the test specimen is estimated as the ratio of the ultimate displacement to yield displacement. As detailed in Table 5, the estimated ductility ratios in both directions and their average are compared with those of the non-retrofitted specimen. The ductility ratios of the specimens retrofitted with 3D-TRM panels and FRP sheets significantly increases compared to that of the specimen NRF. This is because ultimate displacements of the retrofitted specimens significantly increase due to the retrofitting effect caused by the higher tensile strength and toughness of the applied 3D-TRM panels and FRP sheets. The estimated average ductility ratios of the specimens 3DRF4 and 3DRF6 are 7.13 and 6.83, respectively, resulting in a significant increase up to 85.16%, compared with that of the specimen NRF. Particularly, the ductility ratio of the specimen 3DRF4 increases by 4.39% compared with that of the specimen 3DRF6 due to the larger amount of fiber per unit area. As expected, specimens CRF and ARF have higher ductility ratios than the specimen NRF. The ductility ratios of specimens CRF and ARF are 9.07 and 9.69, respectively, and thus, the ductility ratio of the specimen retrofitted with the convention FRP sheets increases up to 151.61%, compared with that of the specimen NRF. It is found that the ductility ratio of the specimen with conventional FRP sheets is higher than the specimen with the 3D-TRM panel. This could be inferred that the larger amount of fibers per unit area contributes to the ductile behavior of the test specimen.

### 4.4. Effect on Stiffness Degradation

**Figure 12:**
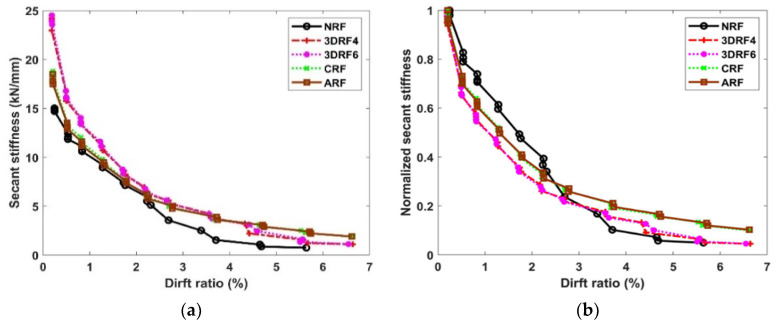
Secant stiffness and drift ratio: (**a**) Secant stiffness; (**b**) Normalized secant stiffness.

As shown in Figure 12a, the secant stiffness is estimated to evaluate the effect of the selected retrofitting method on the stiffness degradation of the test specimen. The secant stiffness normalized by the initial stiffness is also depicted in Figure 12b. Note that the stiffness is monitored up to the drift ratio at the final stage of the experiment. The significant stiffness degradation of the specimen NRF is observed after the drift ratio of about 2% due to the shear-critical behavior. The effect on the stiffness of retrofitted specimens with 3D-TRM panel and conventional FRP sheets shows similar trends. The initial stiffnesses of the specimens 3DRF4 and 3DRF6 specimens retrofitted by 3D-TRM panels increase by 64.10% and 63.56%, respectively, compared with that of the specimen NRF. Those of the specimens CRF and ARF retrofitted by conventional FRP sheets also increase by 22.27% and 20.92%, respectively. The secant stiffness of the specimen retrofitted with 3D-TRM panels is higher than that of the specimen retrofitted by conventional FRP sheets until the maximum lateral force is reached. This is mainly due to the increase of the sectional size along with the 3D-TRM panel thickness. The moment of inertia of the column section with 3D-TRM panels is 46.41% larger than those of other specimens, resulting in significant increase of the initial stiffness of the specimen.

### 4.5. Effect on Energy Dissipation

**Figure 13:**
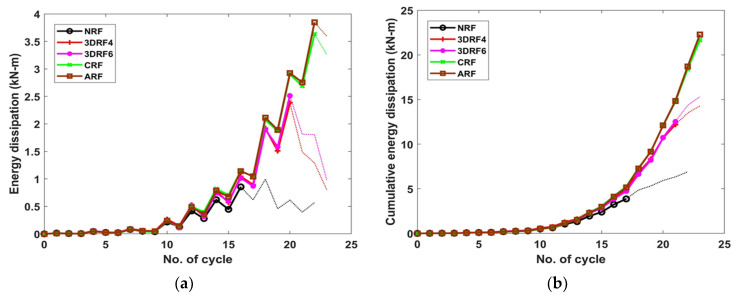
Effect on the energy dissipation: (**a**) Energy dissipation per cycle; (**b**) Cumulative energy dissipation.

The energy dissipation is calculated by taking the area enclosed by the corresponding relationship curve between the lateral force and displacement, and then the cumulative energy dissipation is estimated as the sum of energy dissipation of all cycles. Note that at each drift ratio from 0.25 to 1%, three cycles were repeated, and thereafter only two cycles were repeated for each drift ratio, from 1.5% to failure. As shown in Figure 13, the test with specimen NRF was terminated at 22 cycles due to the rapid reduction of lateral force at post-peak, while other tests were finished at 23 cycles. The energy dissipation per cycle for all specimens is similar until 11 cycles. After then, the dissipated energy of the specimen NRF decreases compared with that of retrofitted specimens due to the yielding and thus slight reduction of the stiffness. In particular, the energy of the specimens retrofitted by FRP sheets steadily increases due to their flexural behavior. Compared to specimen NRF, the cumulative energy dissipations of specimens 3DRF4 and 3DRF6 increase up to 107.10% and 122.09%, respectively. Those of specimens with conventional FRP sheets also increase up to 223.08% compared with the non-retrofitted specimen.

### 4.6. Effect on Strain Distribution

**Figure 14:**
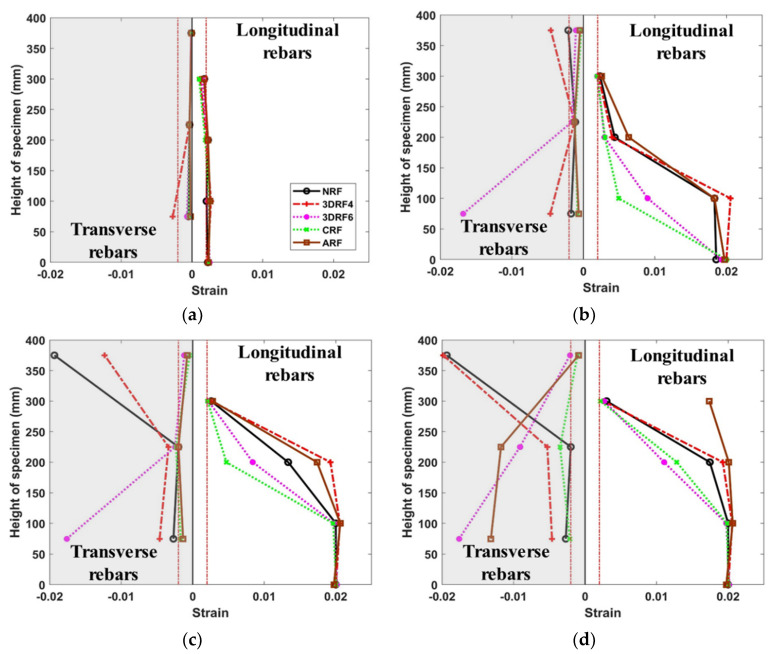
The effect on strain distribution: (**a**) Drift ratio of 1%; (**b**) Drift ratio of 2%; (**c**) Drift ratio of 4%; (**d**) Drift ratio of 6%.

In order to evaluate the effect on the strain distribution of specimens, longitudinal and transverse strains are monitored. Figure 14 shows the maximum strain at each critical cycle. The longitudinal and transverse strains of all specimens did not yield before reaching the drift ratio of 1.0%. At the drift ratio of 1.0%, the transverse strain of the specimen 3DRF4 yielded first, and the longitudinal strain of all specimens also yielded as shown in Figure 14a. Then, the longitudinal strain in the plastic hinge region of all specimens rapidly increased up to 0.02 around the drift ratio of 2%. Figure 14b,d shows that the stirrup strains at the bottom of the specimen NRF are almost the same even if the drift ratio increases. This indicates that the strain at the bottom of the specimen NRF is not properly captured considering the overall performance of the specimens. However, as shown in Figure 14b,c, the transverse strain at the middle of the specimen NRF significantly increased strain after the drift ratio of 2%, where the significant stiffness degradation and a relatively small amount of energy dissipation are observed. Figure 14 also shows the relatively larger transverse strains of the specimens retrofitted with the proposed 3D-TRM panel around at the drift ratio of 4.0%. This indicates that the specimen with the 3D-TRM panel shows flexural behavior before reaching the drift ratio of 4% and afterward shear-critical behavior. The transverse strain at the final stage of the specimen 3DRF6 rapidly increased by 242.27% compared with that of the specimen 3DRF4.

## 5. Conclusions

The paper presents an experimental program to investigate the seismic performance of RC columns retrofitted by a newly developed 3D Textile Reinforced Mortar (TRM). The experimental results are compared with the cases of RC columns with conventional Fiber Reinforced Polymer (FRP) sheets and without any retrofit in terms of the effect on failure mode, hysteretic behavior, ductility ratio, stiffness degradation, energy dissipation, and strain distribution. The most important findings are summarized below.

The flexural capacity of the 3D-TRM panel was evaluated through the four-point flexural test. The flexural strengths of specimens with 3D textiles were increased up to 104.8%, compared to that of the plain specimen. The strength was fluctuated but remained until a significant amount of deflection occurred, indicating the significantly enhanced ductile behavior. The static cyclic tests with RC columns showed that the strength and ductility ratio of RC columns retrofitted by the 3D-TRM panel were improved up to 15.05% and 85.16%, respectively, compared to those of the specimen without any retrofit method. Compared to the specimens strengthened with conventional FRP sheets, the lateral force and initial stiffness of the specimens retrofitted with 3D-TRM panels increased up to 4.81% and 35.71%, respectively. However, the amount of fibers per unit area in the 3D-TRM panel is less than that of the FRP sheets, resulting in lower ductility ratio and energy dissipation. When compared with the plain specimen, the cumulative energy dissipation of specimens with 3D-TRM panels increased up to 122.09%.

Therefore, taking into account the observations from the experimental program described above, it is clearly shown that the proposed retrofitting method with the developed 3D-TRM panel can improve the performance of seismically vulnerable RC columns.

## Figures and Tables

**Table 1:** Mechanical properties of selected textiles (KS K 0521).

Reference Name	Type of Yarn	Type of Textile	Weight (g/yd)	Mesh Size (Length × Width × Height)	Tensile Strength (N)		Elongation (%)	
					Warp	Weft	Warp	Weft
3DTH4	Meta aramid 20/2 + Polyarylate 1500D	Honey comb (3D)	666	18 × 10 × 4 mm	2800	320	47.5	94.9
3DTH6			399	25 × 12 × 6 mm	2800	87	29.4	73.4
Carbon	UHMWPE ^(1)^ 1500D + Carbon 3K	Woven (2D)	490	-	8100	4200	22.8	4.4
Aramid	Vectran 1500D + Meta aramid 20/2		415	-	6600	1600	10.8	37.0

^(1)^: Ultra high molecular weight polyethylene.

**Table 2:** Flexural test of 3D-TRM panel.

Reference Name	Textiles	Mortar Strength (MPa)	Size (mm)	No. of Specimens
NFS	-	43.1	400 × 100 × 30	3
4FS	3DTH4			
6FS	3DTH6			

**Table 3:** Details of test specimens.

Reference Name	Retrofitting Type	Concrete Compressive Strength	Longitudinal Rebar Ratio	Volumetric Ratio of Transverse Rebar
NRF	-	24.7 MPa	2.53% (8-D13)	0.51% (D6@150 mm)
3DRF4	3D-TRM panel with 3DTH4			
3DRF6	3D-TRM panel with 3DTH6			
CRF	Carbon FRP sheets			
ARF	Aramid FRP sheets			

**Table 4:** Measured lateral forces.

Reference Name	Push Direction		Pull Direction		Average Force (kN)	Effect on Lateral Force (%)
	Drift Ratio (%)	Maximum Force (kN)	Drift Ratio (%)	Maximum Force (kN)		
NRF	2.40	79.12	2.09	80.00	79.56	-
3DRF4	3.68	90.56	2.62	92.51	91.54	15.05
3DRF6	3.56	95.40	2.59	85.12	90.26	13.45
CRF	4.71	86.92	3.61	88.33	87.63	10.14
ARF	4.81	88.99	3.72	85.69	87.34	9.78

**Table 5:** Ductility ratio.

Reference Name	Push Direction			Pull Direction			Average Ductility $\left(\mu_{avg}\right)$	Effect on Ductility Ratio (%)
	${\Delta_{y}}^{\;(1)}$	${\Delta_{u}}^{\;(2)}$	$\mu^{\;(3)}$	$\Delta_{y}$	$\Delta_{u}$	μ		
NRF	4.72	18.48	3.92	4.05	15.34	3.79	3.85	-
3DRF4	3.69	28.65	7.76	3.83	24.89	6.50	7.13	85.16
3DRF6	4.14	29.82	7.20	4.12	26.63	6.46	6.83	77.42
CRF	4.28	40.00	9.35	4.28	37.61	8.79	9.07	135.41
ARF	4.00	39.93	9.98	3.94	37.03	9.40	9.69	151.61

^(1)^: yield displacement (mm). ^(2)^: ultimate displacement (mm). ^(3)^: ductility ratio (=$\Delta_{u}/\Delta_{y}$)

## Data Availability

The data presented in this study are available on request from the corresponding author. The data are not publicly available due to privacy.
